# Experimental Investigation of Discharge Phenomena in Electrochemical Discharge Machining Process

**DOI:** 10.3390/mi14020367

**Published:** 2023-01-31

**Authors:** Weidong Tang, Yuhao Zhu, Xiaoming Kang, Cong Mao

**Affiliations:** 1 College of Automotive and Mechanical Engineering, Changsha University of Science and Technology, Changsha 410114, China; 2 School of Mechanical Engineering, State Key Laboratory of Mechanical System and Vibration, Shanghai Jiao Tong University, Shanghai 200240, China

**Keywords:** electrochemical discharge machining, high−speed imaging, discharge, discharge current

## Abstract

Electrochemical discharge machining (ECDM) is a promising non−traditional processing technology used to machine non−conductive materials, such as glass and ceramic, based on the evoked electrochemical discharge phenomena around the tool electrode. The discharge in ECDM is a key factor that affects the removal of material. Moreover, the discharge current is an important indicator reflecting the discharge state. However, the discharge characteristics remain an open topic for debate and require further investigation. There is still confusion regarding the distinction of the discharge current from the electrochemical reaction current in ECDM. In this study, high−speed imaging technology was applied to the investigation of the discharge characteristics. By comparing the captured discharge images with the corresponding discharge current, the discharge can be classified into three types. The observations of the discharge effect on the gas film indicate that a force was exerted on the gas film during the discharge process and the shape of the gas film was changed by the force. In addition, the energies released by different types of discharge were calculated according to the voltage and current waveforms. The discharge frequency was found to increase with the increase in applied voltage and the frequency of the second type of discharge was approximately equal to that of the third type when the applied voltage was higher than 40 V.

## 1. Introduction

Recently, electrochemical discharge machining (ECDM) has been proposed as a potential technology for processing non−conductive materials, such as glass, quartz and ceramics [1]. These materials have a broad range of applications in the optical and semiconductor industries. However, glass, quartz and ceramics have poor machinability because of their hardness and brittleness. It is difficult to obtain good surface qualities and high−aspect ratio microstructures using conventional machining methods. ECDM technology can overcome the limitations and process such non−conductive brittle materials in a precise and efficient manner [2,3,4,5].

ECDM is a non−traditional processing method that is based on the electrochemical discharge phenomenon around the tool electrode [1]. The tool electrode (cathode) and auxiliary electrode (anode) are both partly immersed in an appropriate electrolyte solution (typically an alkali electrolyte solution, such as sodium or potassium hydroxide). Both electrodes are connected to constant direct current (DC) or pulsed voltage sources. The workpiece, dipped in the electrolyte solution, is placed under the tool electrode. The electrolysis reaction begins once a voltage is applied between the two electrodes. Gas bubbles are produced around the tool electrode. When the voltage exceeds the critical value, the high−density gas bubbles will coalesce into a gas film, isolating the tool electrode from the electrolyte [6]. An electric field strength of the order of 107 V/m is generated across the gas film [7]. A discharge then occurs between the tool electrode and the electrolyte [8]. The heat generated by these discharges results in material removal by thermal melting and thermal assist etching if the workpiece is positioned in the vicinity of the electrode [1].

ECDM is a complex process that includes gas film formation and discharge generation [9,10]. The discharge in ECDM is the source of energy release that leads to material removal. The gas film, which serves as the dielectric medium, is a prerequisite for discharge generation. Wüthrich et al. found that the efficiency of the electrical discharge in ECDM mainly depends on the stability of the gas film structure [11]. Many efforts have been devoted to the investigation of the relationship between the gas film structure and the characteristics of discharge performance. Fascio et al. [12] compared the photographs of gas film with scanning potential and divided the voltage–current plots into five regimes. Subsequently, Fascio et al. [13] clarified the onset of the discharge activity based on the analysis of the voltage–current plots. The discharge also plays an important role in the machining efficiency and quality. Ziki [14] found that the rate limiting step changes with the machining depth during the hole drilling process with the use of ECDM as the discharge activity varies with the machining depth. The local glass heating is the rate limiting step at depths that are less than 300 μm. However, at higher depths, hole−flushing becomes the rate limiting step. The machining efficiency decreases with the drilling depth as the hole−flushing becomes more and more difficult. Mailard et al. [15] characterized the micro−holes on glass after gravity−feed drilling and found that four different contours were formed owing to the variations in discharge energy density and electrolyte circulation. They also concluded that a trade−off between the machining efficiency and quality must be made for a given application. Tang et al. [16] proposed the use of a diamond−coated, side−insulated electrode for concentrating the discharge activity on the electrode tip to enhance the machining quality by sacrificing machining efficiency.

As the discharge plays a critical role in ECDM, many efforts have been devoted to understanding the nature and mechanisms of discharge generation. Crichton and McGough [17] found that discharge occurs in the gas film, owing to the growth of layers with low ionic concentrations near the electrodes and to the local variations in the electrolyte flow patterns. Basak and Ghosh [18] proposed that discharging could be modeled as a switching−circuit and the critical current and voltage required to initiate the discharge were estimated. Jain et al. [7] treated each gas bubble as a valve, which generated an arc discharge after its break down due to a high electric field. Kulkarni et al. [19] studied the material removal mechanism based on the observations of time varying current measurements and found that the discharge is a discrete phenomenon. Kang and Tang [20] characterized the different discharges in machining of different material layers in drilling ceramic−coated superalloys according to the different current signals. Jiang et al. [21] considered that the sudden current rise represents the spark generation and estimated the spark energy based on a stochastic model. However, the nature of the discharge in ECDM still remains unclear. There are controversies about whether the discharge is a spark or an arc. Some researchers used the term ‘electrochemical discharge machining’, whereas others referred to it as ‘electrochemical arc machining’, as reported by Wüthrich [1]. Regarding the current waveforms in ECDM, it is difficult to distinguish the discharge current from the electrochemical reaction current since both the discharge activity and electrochemical reaction take place.

In this study, a high−speed camera was applied to capture the discharge phenomenon around the tool electrode tip. The captured discharge images were compared with the corresponding current waveforms, and different types of discharges were then classified based on the discharge currents. The energies released by the different discharges in ECDM were calculated and their frequencies were estimated.

## 2. Materials and Methods

### 2.1. Experimental Setup

**Figure 1:**
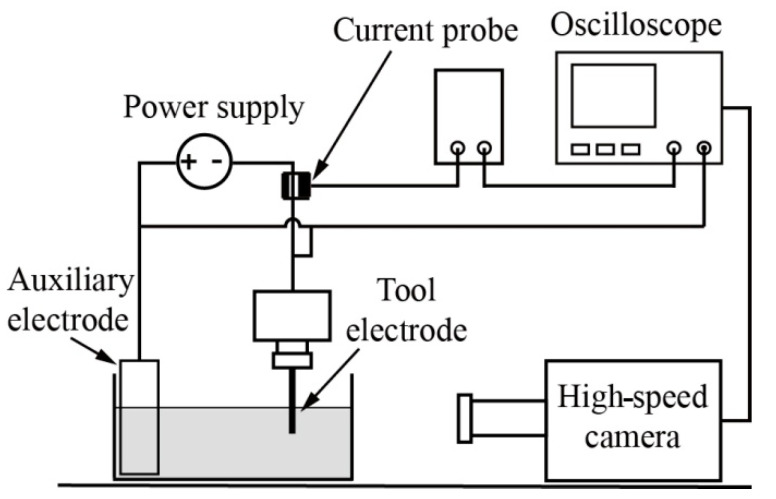
Experimental setup for capturing the discharge phenomenon and the gas film formation.

Figure 1 shows the experimental setup for capturing the gas film and discharge around the tool electrode using a high−speed camera during the electrochemical discharge process. The tool electrode and the auxiliary electrode were connected by a DC power supply. Both electrodes were semi−immersed in the electrolyte. The electrolytic cell was made of poly (methyl methacrylate) given its transparent properties, which are beneficial for high−speed imaging. When a preset voltage was applied, both the voltage and current of the electrochemical discharge process were collected by an oscilloscope, wherein the current was collected through a current probe based on the Hall effect. Once the oscilloscope detected the current, it triggered the high−speed camera in real time and the high−speed imaging began. The experimental parameters for the electrochemical discharge high−speed imaging are shown in Table 1.

### 2.2. Tool Electrode

For the general cylindrical tool electrode, the discharge location was random. The discharge may occur at the bottom of the tool electrode or on the side wall of the tool electrode. In order to clearly capture the image of the discharge, it is necessary to fix the discharge to a point in the visible region. The conical electrode can concentrate the discharge at the tip of the tool electrode, ensuring that each discharge can be captured. Therefore, the conical electrode was used in this paper.

**Figure 2:**
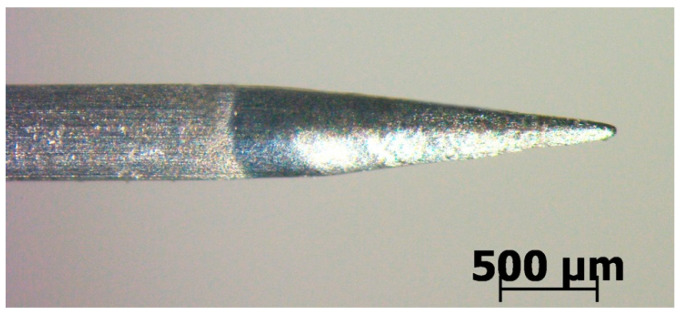
Tool electrode.

Figure 2 shows the tool electrode used in the high−speed imaging experiments. This conical electrode was made by electrochemical machining (ECM) using sodium hydroxide as the electrolyte. Duong et al. [22] had mentioned that different electrode shapes can be achieved by controlling the experimental parameters in ECM. The diameter of the electrode was 500 μm and the fillet radius at the electrode tip was 30 μm. The material of the electrode was tungsten, which is extensively used in ECDM owing to its high−melting point and good electrical conductivity properties, as reported by Mousa et al. [23] and Yang et al. [24]. Jiang et al. [21] indicated that the discharge occurs at the tip of the conical electrode where the maximum electric field strength is located. Thus, using the conical electrode in the experiments helped to increase the consistency of discharge generation and facilitated the capture of the discharge phenomenon by the camera.

The electrolyte used in the experiments was sodium hydroxide with a concentration of 6 mol/L. The power supply was a smooth DC power with an applied voltage in the range of 30 V and 40 V. The conical electrode used in this study is small. When the applied voltage is higher than 30 V, the time for the gas film formation is very short, which makes it difficult to capture the evolution phenomena of the gas film using the camera. In order to obtain the complete evolution of the gas film formation process around the tool electrode, a voltage of 30 V was applied. A relatively high voltage (40 V) was used in the experiment to capture the discharge around the electrode tip since bright discharges can be produced under this condition benefiting the capture of obvious discharge images. The camera parameters were set before the experiments by adjusting the time interval between any two adjacent images and the exposure time. Once the power is applied, the camera, triggered by the real−time current signal, begins to capture the gas film and discharge images.

## 3. Results and Discussion

### 3.1. Gas Film around the Conical Electrode

**Figure 3:**
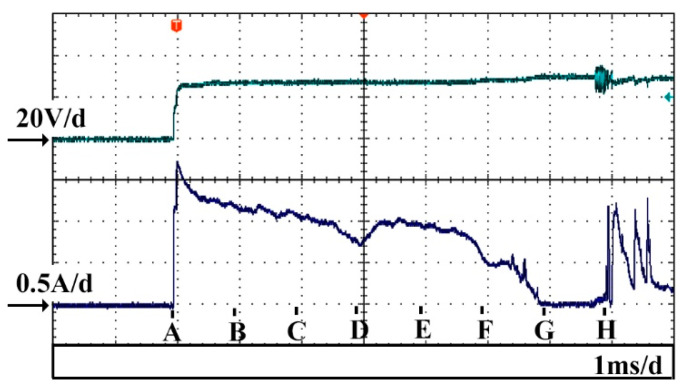
Temporal variations of voltage and current waveforms during gas film formation.

**Figure 4:**
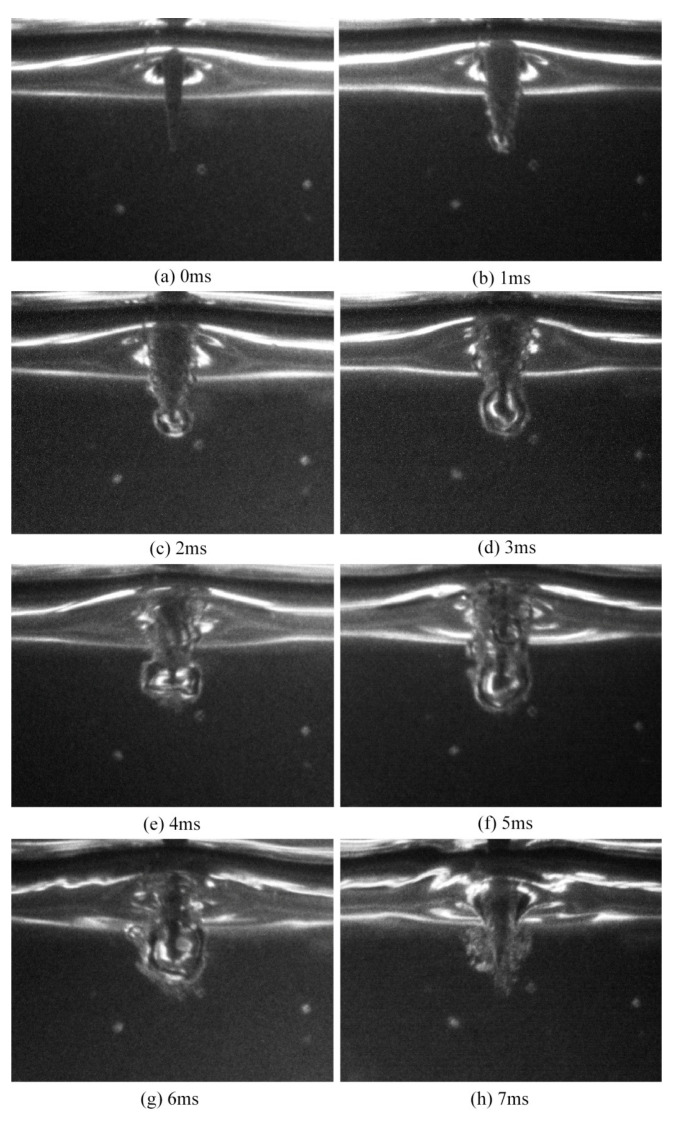
Images of bubbles produced around the conical electrode at different times. (**a**) 0 ms, (**b**) 1 ms, (**c**) 2 ms, (**d**) 3 ms, (**e**) 4 ms, (**f**) 5 ms, (**g**) 6 ms, (**h**) 7 ms.

Figure 3 and Figure 4 show the voltage and current waveforms and the corresponding bubble generation phenomena around the conical electrode with the applied voltage of 30 V, respectively. In order to capture the bubble generation phenomenon from the initial moment when the voltage was applied, a step voltage was used in the experiment as shown in Figure 3. Point A in Figure 3 is the starting moment for the imaging acquisition. The images in Figure 4 were captured by the high−speed camera with a time interval of 1 ms.

Figure 4a shows the image at the moment the voltage had just been applied. At this instant, the bubbles around the electrode were not obvious. The corresponding current signals are shown in Figure 3. As can be observed from the figure, the current reached the peak of 1.7 A at the moment the voltage was applied. Subsequently, the current curve exhibited a downward trend during the following 6 ms, and the corresponding bubble generation phenomena around the conical electrode are shown in Figure 4b–g. It can be observed from the images that the volume of the bubbles around the electrode was continuously increasing. Bubble convergence also occurred at the same time, especially at the tip of the electrodes, thereby resulting in one big bubble. This bubble became larger since it merged with the nearby bubbles. During this process, the chemical reaction current became progressively smaller and it eventually decreased to almost 0 mA at 6 ms after the power was applied. It can be observed in Figure 4g that at this moment, the bubbles around the electrode merged into one big bubble, isolating the electrode from the electrolyte. Thus, the electrochemical reaction stopped and the current decreased to nearly 0 mA. Subsequently, the big bubble moved upwards under buoyancy and eventually formed a complete gas film around the electrode, as shown in Figure 4h. The gas film was unstable at this moment due to the inertia of the moving bubble. Therefore, the unstable gas film contacted the tool electrode and the electrochemical reaction currents were produced, as shown in the current waveforms after point H in Figure 3. After forming a stable gas film around the electrode, it will enter the discharge stage.

### 3.2. Discharge in ECDM

**Figure 5:**
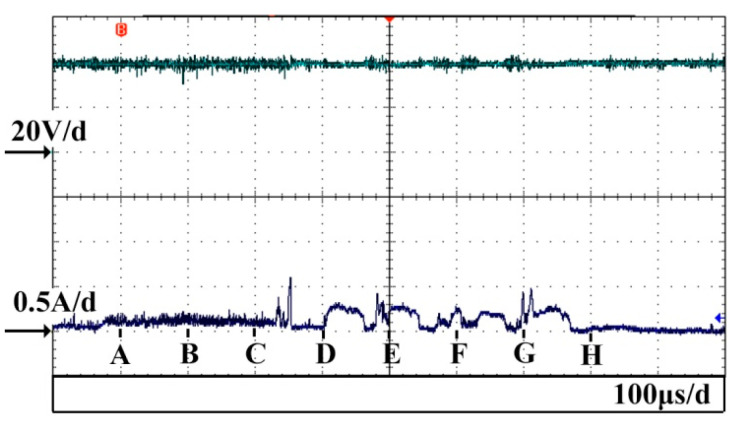
Voltage and current waveforms during the discharge stage.

**Figure 6:**
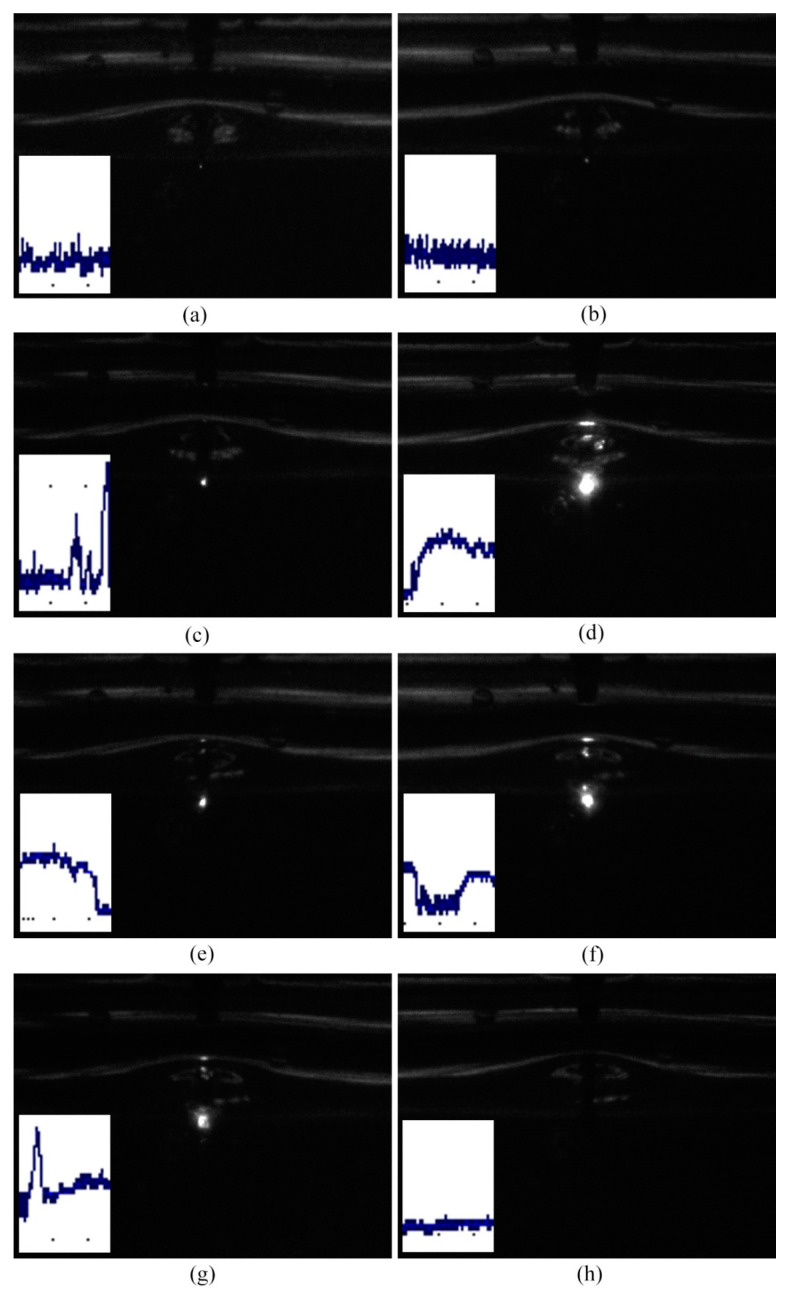
Discharge images and the corresponding discharge current signals in ECDM at different times. (**a**) 0 μs, (**b**) 100 μs, (**c**) 200 μs, (**d**) 300 μs, (**e**) 400 μs, (**f**) 500 μs, (**g**) 600 μs, (**h**) 700 μs.

Figure 5 shows the voltage and current waveforms at the discharge stage in ECDM. In order to make the brightness of the discharge more obvious, thus benefiting the capturing of the discharge images by the high−speed camera, the applied voltage was set to 40 V. Figure 6 displays the discharge images at time intervals of 100 μs; the corresponding current signals for each image are shown at the bottom left of the image. Point A in Figure 5 is the starting moment for the imaging acquisition. The exposure time for each image is 50 μs. In order to improve the quality of the discharge images, a weak light was used in the experiments.

Since each image was generated within a time period of 50 μs, the corresponding current for Figure 6a is the current generated at the 50 μs duration immediately after point A in Figure 5. It can be observed from this current waveform that numerous, low−intensity, narrow pulses are distributed on the current curve which oscillates within the range of 100–200 mA. The corresponding current for Figure 6b is similar to the current in Figure 6a. It can be observed from the discharge images in Figure 6a,b that both discharges are very weak. Thus, it can be inferred that the discharge can occur for discharge currents in the range of 100–200 mA, even though the discharge brightness is very low.

Three pulses appear in the discharge current for Figure 6c. The peak values for the three current pulses are 380 mA, 220 mA and 600 mA, respectively, and the corresponding pulse durations are 8 μs, 4 μs and 10 μs. It can be observed from the image in Figure 6c that an electrical discharge took place at the tip of the tool electrode, thus verifying that the current pulses in Figure 6c are discharge currents. These discharge currents are larger than those in Figure 6a,b. Thus, the discharge in Figure 6c is relatively brighter compared with Figure 6a,b. The larger the discharge current, the more energy is released, thereby resulting in a brighter discharge.

The corresponding current in Figure 6d shows that the current had increased from 40 mA to 300 mA. More details are shown at point D in Figure 5. As can be observed, the leap current was maintained at the level of 300 mA for 50 μs and the entire pulse width of the leap current was 60 μs. Subsequently, the current dropped to 40 mA again. Because of this leap current, the discharge at the electrode tip in Figure 6d is particularly brighter as compared with Figure 6a–c.

The current also increased in Figure 6e–g as compared with Figure 6a–c, and the corresponding discharges are thus brighter. It can be observed from the current in segment EH in Figure 5 that there are four leap currents occurring in this segment and their peak values are located within the range of 200–300 mA. The pulse durations for each leap current were 45 μs, 20 μs, 50 μs and 90 μs, respectively. It can also be seen that two short pulse currents with peak values of 400 mA and 500 mA occurred at point G in Figure 5.

After these several leap currents, the current dropped to 40 mA. As shown in Figure 6h, no discharge appeared at the electrode tip at this moment. As can be observed from the current curve after point H in Figure 5, the current dropped to almost 0 mA.

**Figure 7:**
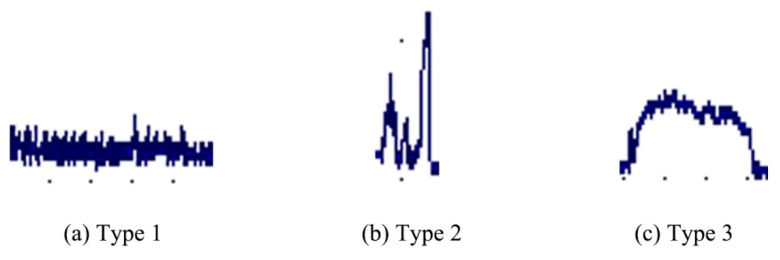
Three different types of discharge current signals in ECDM. (**a**) Type 1 is a current with a small amplitude and a narrow pulse duration, (**b**) type 2 is a high−intensity current with a short pulse duration and (**c**) type 3 is a high−intensity current with a long pulse duration.

According to the above analysis, the discharge currents in ECDM can be classified into three categories, as shown in Figure 7. The first type is shown in Figure 7a, whereby the discharge current presents the characteristics of a small current with narrow pulse duration, and with the current values ranging from 100 mA to 200 mA. In addition, numerous small pulses are distributed on the current curve. The pulse width of the small pulse can be measured to be 1 μs by amplifying the current curve. The corresponding discharge of this current is very weak, as shown in Figure 6a,b.

The second type of discharge current is shown in Figure 7b. It has the characteristics of a high current intensity and short pulse duration. The peak current of this type is in the range of 200–600 mA and the pulse width is approximately 8 μs. The discharge corresponding to this type of current was stronger than the first one and the spark was brighter. 

Figure 7c shows the third type of discharge current; it is characterized by a high current intensity and long pulse duration. The peak current of this type is in the range of 200–300 mA and the pulse duration ranges from 20 μs to 90 μs. 

As described in previous study, a spark is a sudden transient and noisy discharge between two electrodes, and an arc shows a stable thermionic phenomenon [17]. Therefore, discharge can be distinguished according to the discharge current and the light emitted. If the discharge current shows a relatively stable state, which means the discharge current remains at a stable value over a period of time (such as tens of microseconds), and furthermore, the corresponding discharge light is bright, then these kinds of discharges can be considered arcs. On the other hand, if the discharge current shows a sudden transient and noisy characteristic, along with a weak discharge light, the discharges can be considered sparks. Therefore, the third type of discharge can be seen as an arc and it is the brightest. The first and the second types of discharge can be seen as sparks. Therefore, both sparks and arcs occur during the ECDM process. This finding is consistent with the findings reported by Crichton and McGough [17].

### 3.3. Energy Released by the Discharge

The discharges in ECDM were categorized into three types in the previous section. Based on the voltage and current curves, the energy released by each type of discharge can be calculated in accordance with the following formula:

(1) E=UIT

where *E* is the discharge energy, *U* is the discharge voltage, *I* is the discharge current and *T* is the discharge time. According to the literature [25], the discharge voltage *U* between electrodes can be regarded as the voltage applied between the tool electrode and the auxiliary electrode. It can be observed from Figure 5 that the discharge voltage between the electrodes can be considered constant. It was set to 40 V in this article. As can be observed from the current curve, the discharge current varies with time. Thus, Equation (1) can be expressed as

(2) $$E=U\int_{0}^{t_{0}}i\left(t\right)dt$$

where *i*(t) is the time varying discharge current and *t*_0_ is the discharge time which can be obtained from the discharge current curve. Since the discharge energy of the first type of discharge is very small and has a minor contribution to the material removal, this article focuses on the calculation of the discharge energies of the second and the third types. According to Equation (2), the discharge energy of the second type can be calculated to be in the range of 1.76 × 10^−5^ J–1.2 × 10^−4^ J and the discharge energy of the third type is within the range of 2.0 × 10^−4^ J–7.2 × 10^−4^ J.

The total amount of electric charge released per discharge can be obtained from the following formula:

(3) $$Q=\int_{0}^{t_{0}}i\left(t\right)dt$$

where *Q* is the total electric charge, *i* is the time varying discharge current and *t*_0_ is the discharge time. According to Equation (3), the total amount of electric charge released by the second type of discharge can be calculated. This is found to be in the range of 4.4 × 10^−7^ C–3.0 × 10^−6^ C, while the total amount of electric charge released by the third type of discharge is in the range of 5.0 × 10^−6^ C–1.8 × 10^−5^ C.

According to the total amount of electric charge, the number of electrons participating in the discharge can be obtained using the following formula:

(4) $$n=\frac{Q}{\mathrm{NA}*e}$$

where *n* is the number of moles of electrons involved in the discharge, *Q* is the total electric charge released by one discharge, NA is Avogadro’s number and *e* is the electric charge carried by one electron. According to Equation (4), the number of electrons participating in the second type of discharge can be calculated to be in the range of 4.6 × 10^−12^ mol–3.1 × 10^−11^ mol and for the third type of discharge, the number of electrons is calculated to be in the range of 5.2 × 10^−11^ mol–1.87 × 10^−10^ mol.

### 3.4. Effects of Discharge on Gas Film

According to the above analysis, the energy released by the third type of discharge is the largest among the three studied forms. When the discharge is generated by the breakdown of the gas film, a force is produced which will change the shape of the gas film and further affect the current signal.

**Figure 8:**
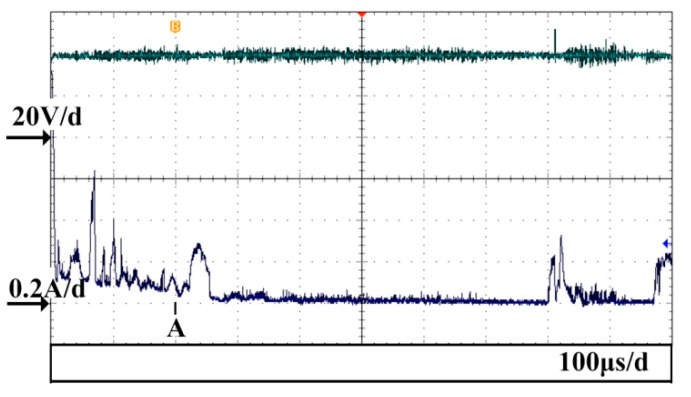
Voltage and current waveforms including a third type of discharge.

**Figure 9:**
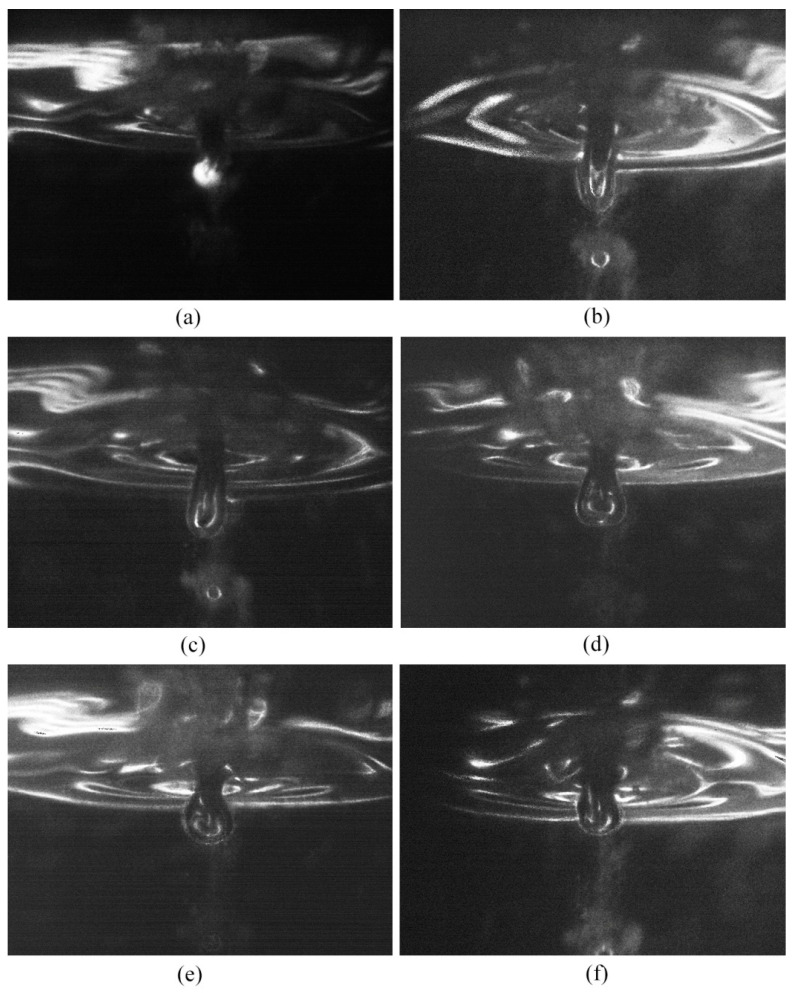
Effects of type−3 discharge on gas film. (**a**) 0 μs, (**b**) 100 μs, (**c**) 200 μs, (**d**) 300 μs, (**e**) 400 μs, (**f**) 500 μs.

Figure 8 shows the current waveform that also includes a third type of discharge which occurs at point A in the figure. Point A is the starting point of the image capturing process. Figure 9 displays the corresponding discharge and gas film images, and the time interval between adjacent images is 100 μs. The image corresponding to the third type of discharge is shown in Figure 9a. As observed, there is a bright discharge at the tip of the electrode. The current dropped to values within the range of 0–40 mA when the discharge was completed, as shown in Figure 8. At this time, the corresponding gas film morphology is shown in Figure 9b–f. As can be observed from the images, the gas film around the tool electrode was lengthened downwards, owing to the force produced by the third type of discharge. A big oval bubble was formed around the tip of the electrode. It can be measured using Figure 9b that the bubble was lengthened by 170 μm in the vertical direction.

It can be concluded from the above phenomenon that a force will is on the electrolyte during the discharge process that thus changes the shape of the gas film. For the third type of discharge, the effect of the discharge on the gas film is obvious. The shapes of the gas film in Figure 9b,c indicate that the direction of the force exerted on the electrolyte is vertically downwards. This direction is consistent with the direction of the arc produced in the gas film. The arc column is vertical, beginning at the electrode tip and ending at the electrolyte under the electrode tip. A previous study [17] observed that the discharge arc in ECDM is located between the anode and the cathode. In the discharge arc column, positive ions move to the cathode (the tool electrode) and anions move to the anode (the electrolyte). Thus, the electrolyte was forced to move downwards owing to the bombardment of the anions, resulting in the expansion of the gas film. As can be observed in Figure 9b,c, a circular bubble was produced below the oval gas film. This is owing to the fact that the bombardment of anions forced the local gas film under the electrode tip to escape from the main gas film, thus generating a circular bubble. The main gas film was dragged into an oval shape owing to the downward movement of the circular bubble.

The big oval gas film moves upwards owing to the buoyancy, as shown in Figure 9d–f. The current is small during this process. It can be observed in Figure 8 that the current is only within the range of 0–40 mA, thereby indicating that no discharge took place during this process. This small current could be an electrochemical reaction current with low intensity that occurs during the upward movement of the gas film. When the gas film moves further up and forms a complete and stable gas film, the discharge occurs again, as shown in Figure 8.

### 3.5. Statistics of Different Discharges in ECDM

**Figure 10:**
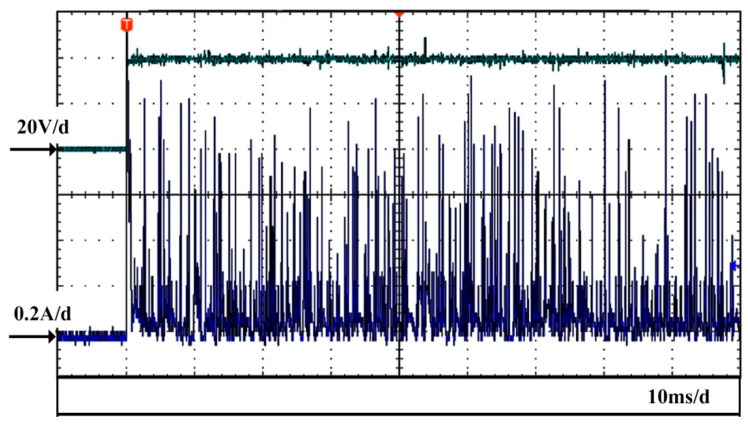
Voltage and current signals within a time period of 90 ms.

In order to count various discharge types in ECDM, the ECDM current was recorded over a prolonged time period. Figure 10 shows the voltage and current curves recorded within a period of 90 ms at an applied voltage of 40 V. Five records were collected to increase the total number of samples. Statistics were estimated based on the classification of discharges in Figure 7. Since the current pulse of the first type of discharge was too small to count, we mainly focus here on the statistics of the second and the third types of discharge.

The pulse current with a peak value around 1 A in Figure 10 is the electrochemical reaction current that indicated that the gas film was disturbed or destroyed during the discharge process. Therefore, the contact between the electrode and the electrolyte ignited the electrochemical reactions. According to the previous analysis, the peak currents of the second and the third types of discharge are in the range of 200–600 mA. The statistics estimated from the current curves within the time period of 450 ms in the five records collected herein reveal that the second type of discharge occurred 835 times, while the third type of discharge occurred 1320 times. Thus, the frequencies of the second and the third types of discharges were 1856 Hz and 2933 Hz, respectively. When the gas film is disturbed or broken, an electrochemical reaction takes place and the corresponding electrochemical reaction current is high. The current that is higher than 700 mA is assumed to be the electrochemical reaction current, and the frequency of the electrochemical reaction is 690 Hz during the discharge stage. Thus, the average gas film lifetime is 1.45 ms and the average numbers of discharge occurrences of the second and third types during the gas film life period are 2.7 times and 4.3 times, respectively.

**Figure 11:**
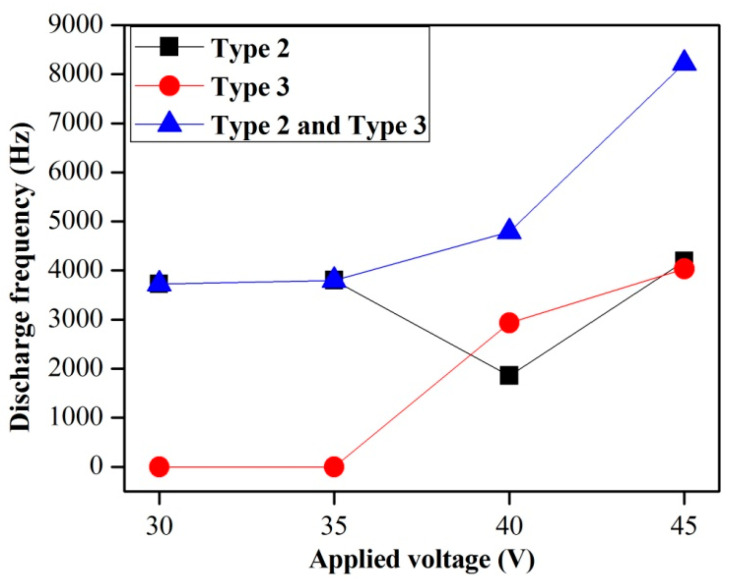
Discharge frequencies for different applied voltages.

Figure 11 shows the discharge frequencies at different applied voltages. It can be seen that the total frequencies of the second and the third types of discharge increase at increasing applied voltages. This explains why the material removal rate increases at increasing voltages. The frequency of the third type of discharge increased from 0 Hz to 2.9 kHz when the applied voltage increased from 35 V to 40 V, while the frequency of the second type of discharge dropped from 3.8 kHz to 1.8 kHz, thus indicating that the third type of discharge occurred only when the applied voltage is high enough. The lowest voltage for the occurrence of the third type of discharge was 38.5 V in this study. The discharge frequency curves also revealed that the appearance of the third type of discharge reduces the possibility of occurrence of the second type of discharge. The frequency of the second type of discharge is approximately equal to that of the third type when the applied voltage is higher than 40 V.

## 4. Conclusions

A high−speed imaging technology was employed to investigate the characteristics of the discharge in ECDM. The captured discharge images were compared with the real−time discharge current. The following conclusions can be drawn:

(1) The discharge can be classified into three types according to the brightness of the discharge images and the corresponding discharge current. The first type of discharge presented the characteristics of a low−amplitude current with a narrow pulse duration and with current intensity values in the range of 100 mA to 200 mA. The pulse duration was 1 μs and the discharge was very weak. The second type of discharge showed the characteristics of a high−intensity current and short pulse duration. The peak current of this type was in the range of 200–600 mA and the pulse width was approximately 8 μs. The discharge brightness was stronger than the first type. The third type of discharge was characterized by a high−intensity current and long pulse duration. The peak current of this type of discharge was in the range of 200–300 mA and the pulse duration ranged from 20 μs to 90 μs. The third type of discharge resembled an arc and it was the brightest.
(2) The observations of the effect of discharge on the gas film indicate that a force was exerted on the electrolyte during the discharge process and that the gas film was dragged into a large oval bubble owing to the bombardment of anions on the electrolyte. Meanwhile, the current dropped to almost 0 mA during the deformation process of the large oval bubble.
(3) The energies of different types of discharges were calculated according to the voltage and current waveforms. The discharge energy of the second type was calculated to be in the range of 1.76 × 10^−5^ J–1.2 × 10^−4^ J, and the discharge energy of the third type was in the range of 2.0 × 10^−4^ J–7.2 × 10^−4^ J. Experimental results showed that the discharge frequency increased with increasing applied voltages and the third type of discharge occurred only when the applied voltage was high enough. In addition, it was found that the frequency of the second type of discharge was approximately equal to that of the third type when the applied voltage was higher than 40 V.

## Figures and Tables

**Table 1:** Experimental parameters of electrochemical discharge high−speed imaging.

Factors	Parameters
Tool electrode polarity	Cathode
Tool electrode	Conical electrode
Tool electrode material	Tungsten
Tool electrode immersion depth	1 mm
Electrolyte	6 mol/L NaOH
Applied voltage	30 V, 40 V
Frame rate	10,000 fps

## Data Availability

Not applicable.
